# Implementation of a Multidisciplinary Guideline for Low Back Pain: Process-Evaluation Among Health Care Professionals

**DOI:** 10.1007/s10926-016-9673-y

**Published:** 2016-10-03

**Authors:** Arnela Suman, Frederieke G. Schaafsma, Rachelle Buchbinder, Maurits W. van Tulder, Johannes R. Anema

**Affiliations:** 1 Department of Public and Occupational Health, EMGO+ Institute for Health and Care Research, VU University Medical Centre, PO Box 7057, 1007 MB Amsterdam, The Netherlands; 2 Research Centre for Insurance Medicine, Collaboration Between AMC-UMCG-UWV-VUmc, Department of Public and Occupational Health, VU University Medical Centre, PO Box 7067, 1007 MB Amsterdam, The Netherlands; 3 Monash Department of Clinical Epidemiology, Cabrini Institute and Department of Epidemiology and Preventive Medicine, Monash University, Suite 41, Cabrini Medical Centre, 183 Wattletree Rd, Melbourne, VIC 3144 Australia; 4 Department of Health Sciences, Faculty of Earth & Life Sciences, VU University Amsterdam, De Boelelaan 1085, 1081 HV Amsterdam, The Netherlands

**Keywords:** Multifaceted implementation strategy, Low back pain, Guideline implementation, Process evaluation, Healthcare professionals

## Abstract

*Background* To reduce the burden of low back pain (LBP) in the Netherlands, a multidisciplinary guideline for LBP has been implemented in Dutch primary care using a multifaceted implementation strategy targeted at health care professionals (HCPs) and patients. The current paper describes the process evaluation of the implementation among HCPs. *Methods* The strategy aimed to improve multidisciplinary collaboration and communication, and consisted of 7 components. This process evaluation was performed using the Linnan and Steckler framework. Data were collected using a mixed methods approach of quantitative and qualitative data. *Results* 128 HCPs participated in the implementation study, of which 96 participated in quantitative and 21 participated in qualitative evaluation. Overall dose delivered for this study was 89 %, and the participants were satisfied with the strategy, mostly with the multidisciplinary approach, which contributed to the mutual understanding of each other’s disciplines and perspectives. While the training sessions did not yield any new information, the strategy created awareness of the guideline and its recommendations, contributing to positively changing attitudes and aiding in improving guideline adherent behaviour. However, many barriers to implementation still exist, including personal and practical factors, confidence, dependence and distrust issues among the HCPs, as well as policy factors (e.g. reimbursement systems). *Conclusions* The data presented in this paper have shown that the strategy that was used to implement the guideline in a Dutch primary care setting was feasible, especially when using a multidisciplinary approach. However, identified barriers for implementation have been identified and should be addressed in future implementation.

## Background

Implementing health care innovations or guidelines is a complex and challenging task [1], which likely can influence the effects of implementation strategies to a great extent [2, 3]. Studies evaluating implementation strategies designed to increase the uptake of health care innovations and/or guidelines into practice should ideally evaluate the implementation process. Process evaluations of implementation strategies are useful for various purposes. For example, they may provide insight into why certain strategies succeed or fail to lead to desired and effective changes in health care practice and patient care. Studying determinants of success or failure can help explain heterogeneous effects of different implementation strategies, and the results of process evaluations can be useful to improve existing strategies or inform the development of more effective implementation strategies in the future [3, 4].

Low back pain (LBP) is one of the most prevalent and costly health care problems worldwide [5]. It has a lifetime prevalence of >70 %, and it is the leading global cause of disability [5–7]. While there have been many attempts to tackle the societal and financial burden of back pain, given the high prevalence and burden of LBP it is obvious that few interventions have succeeded in providing sustained long-term solutions to the problem. For instance, French et al. [8] have attempted to improve general practitioner management of back pain in Australian general medical practice by means of a cluster randomised trial. Their intervention led to small changes in general practitioners’ intention to guideline-adherent behaviour, but did not result in statistically significant changes in actual behaviour, although a process-evaluation of their trial suggested that their intervention was delivered with high levels of adherence to the intervention protocol [9].

In an attempt to reduce the burden of LBP in the Netherlands, in 2010 the ‘Multidisciplinary guideline for nonspecific low back pain’ was developed [10]. Implementation of this guideline in the Amsterdam area, applying a multifaceted, patient- and health care professional-based strategy in a cluster randomised controlled trial (RCT), commenced in 2013. In this implementation study, an interactive multimedia campaign for patients with LBP was combined with continuing medical education (CME) training sessions for health care professionals (HCP). The details of this RCT are described elsewhere [11]. The present study describes the process evaluation of the implementation strategy targeted at HCPs. A process evaluation of the patient-based strategy will be reported elsewhere.

The goals of the current study were: (1) to evaluate whether the implementation strategy was conducted as planned; (2) to assess the feasibility, barriers and facilitators of the multifaceted implementation strategy for the guideline implementation in a primary care setting; (3) to gain insight into the satisfaction and experiences of HCPs with the implementation strategy; and (4) to gain insight into process data in order to help in understanding and interpreting the outcomes of the effect evaluation of this implementation study.

## Methods

This process evaluation was performed alongside a stepped-wedge RCT to test the cost-effectiveness of a multifaceted implementation strategy for the Dutch multidisciplinary guideline for nonspecific LBP. Details of the procedures and methods of the RCT, as well as details on the medical ethical review for this study have been described in more detail elsewhere [11].

### Context

In the Netherlands, approximately 98 % of citizens [12] are registered with a general practitioner (GP), who functions as a gatekeeper for specialised medical care, e.g. hospital-based diagnostics (e.g. MRI), and treatment (e.g. surgery). Reimbursement of consultation fees for specialised medical care is often dependent on referral from a GP or occupational physician (OP). Up to 2006, referral was also necessary for consultation and treatment by physiotherapists (PTs). In 2006, with the introduction of a new health care system in the Netherlands, PTs became accessible without referral. However, PT is not included in the basic public health insurance in the Netherlands, and patients need to contract additional health care insurance in order to obtain PT treatment reimbursement. OPs are usually employed by occupational health services (OHS), which can be hired by larger companies or individual employers to assist in occupational health matters. Some companies may employ OPs themselves, which is often done by larger for-profit businesses. Usually, OPs are only consulted in LBP if the complaints might be work-related, or if the patient/worker is on sick leave for 6 or more weeks. At this time-point the OP is obliged by law to assess the patients’ situation and abilities for work.

### Study Population

Since 2003, The VU University medical centre, department of General Practice and Elderly Care Medicine, is involved in an academic network of GPs in the Amsterdam area. The aim of this network is to collaborate on scientific research and primary care innovation and thus contribute to the development and optimization of family medicine. To recruit GPs, presentations and information about the study were provided at network meetings, newsletters and e-mails. As this study aimed to increase guideline adherence by stimulating and improving multidisciplinary collaboration and communication, the aim was to recruit HCPs that were acquainted with each other or shared patients. Therefore, at the start of the study, participating GPs were asked to provide a list of PT practices they regularly referred LBP patients to. These PT practices received an information package about the trial by postal mail. This was followed with a telephone call to provide additional information about the trial, answer any questions, and to ask whether the PT practice would participate in the trial. OPs in the Netherlands do not regularly collaborate with GPs and PTs. The aim was to recruit OPs that work in the Amsterdam area, in order to increase the possibility of future collaboration. Informative presentations about the trial were held at a meeting for OPs working in this area. During this meeting, OPs could sign up for participation in the trial. In addition, OPs in the network of the department of Public and Occupational Health of the VU University medical centre were informed and invited to participate by e-mail.

Participating HCPs were allocated to one of 4 clusters, which were based on the HCPs’ geographic proximity to each other. All clusters sequentially received the intervention (i.e. multifaceted implementation strategy) and the HCPs were invited to participate in this process evaluation immediately after the cluster they had been allocated to had completed the intervention phase.

### Multifaceted Implementation Strategy

The implementation strategy for HCPs consisted of several components, which are summarized in Box 1 and described in detail below. The components were targeted at the barriers that were identified during the development of the guideline, and based on strategies most effective to change such barriers, as described by Grol and Wensing [4].

**Box 1:** Components of the multifaceted implementation strategy for HCPs

1. A multidisciplinary CME training session
2. Take-home educational material
3. Rules of conduct for communication and collaboration
4. Contact details of participating HCPs
5. Quarterly reminders
6. Monthly newsletters
7. An interactive website and social media

*CME* continuing medical education

*HCPs* health care professionals

#### Multidisciplinary Continuing Medical Education (CME)

The aim of this CME training session was to improve effective multidisciplinary collaboration and communication between the participating HCPs when treating patients with nonspecific LBP, to reduce unnecessary health care utilization, and to improve patient-doctor communication. These were the main recommendations in the guideline, which included referrals, transfer of patient data, supervision of patients, and patient education and treatment. Table 1 displays the learning objectives for this training.

**Table 1:** Learning objectives for the CME training

1. HCPs are able to adequately apply the content of the guideline into practice
2. HCPs are able to identify barriers and solutions for adequate multidisciplinary collaboration and they are able to apply these solutions into practice
3. HCPs are able to identify when, why, and how to collaborate with professionals from other disciplines
4. HCPs are familiar with the requirements for adequate re-/deferral and are able to satisfy these conditions when re-/deferring a patient
5. HCPs are able to work in a multidisciplinary team and come to joint treatment decisions
6. HCPs are able to inform, treat, and re-/defer patients in accordance with the guideline recommendations

*CME* continuing medical education

*HCPs* health care professionals

The training was developed in close collaboration with a professional educationalist and met the educational requirements of the Dutch physicians and physiotherapists associations. All training sessions were organized and given by at least 1 member of the research team (to assure scientific quality), and 1 practicing HCP (to ensure relevance and connection to daily practice). The training sessions lasted 2.5 h each and were divided into 5 components, which will be described in detail below.

1. The training sessions started with a short, plenary lecture that introduced the trial, the guideline, and the need for the implementation of the guideline.
2. To further underline this need, especially the need for multidisciplinary collaboration and communication, a short video (‘FlashBack’) was shown. This award winning video about patient and multidisciplinary communication on LBP management was developed in 2006 for use in medical education in various teaching hospitals in the Netherlands. The video shows a patient case of LBP and the consultations the patient has with several HCPs (GP, OP, neurologist). In this video, the communication between the patient and the various HCPs, as well as the communication among the HCPs is emphasized, and the effects of these communications on patient outcomes are highlighted.
3. Subsequently, HCPs were divided into small interdisciplinary groups in which they worked on 2 assignments. The first assignment was a so-called ‘carousel’ in which barriers for effective communication and collaboration in clinical practice and solutions for these barriers were discussed. The carousel was performed in 3 subsequent rounds. In the first round, the groups discussed barriers and wrote them down. In the second round, the working groups interchanged their written barriers and discussed possible strategies that could help overcome the barriers noted in another working group. In the third round, the groups presented their barriers and how they would cope with them, and formulated strategies for overcoming these barriers.
4. The second assignment for the groups was a role play. A patient case was presented in which interdisciplinary and patient-doctor communication were underlined. Based on this case, the groups conducted a role play where the HCPs switched professions in order to train thinking outside their own reference frames and learn to look from another professional perspective at the LBP patient. This was designed to improve interdisciplinary understanding and collaboration. After the role play, a plenary discussion followed in which the multidisciplinary collaboration and the interaction with the patient were discussed, as well as the applicability of the strategies previously agreed upon for overcoming barriers.
5. The last component of the training session was a discussion about rules of conduct that have to be followed by all HCPs involved in order to achieve effective communication and collaboration in daily practice. The session concluded by having the HCPs pick the 10 most important rules of conduct they considered essential for interdisciplinary communication and collaboration.

#### Take-Home Educational Material

All attending HCPs received take-home educational materials that were used during the training, including the guideline and links to relevant literature (books and websites).

#### Rules of Conduct and Contact Details

Following the training sessions, all HCPs (including those who did not attend the session) received the strategies and rules of conduct they had discussed during the session by e-mail, accompanied by the contact details of all HCPs (including those who did not attend the session) from their cluster.

#### Quarterly Reminders

HCPs received a reminder about the strategies and rules by e-mail, along with the contact details (component 4) and a copy of the guideline directly after the training, and again after 3, 6, and 12 months.

#### Monthly Newsletters

Monthly newsletters were sent to all participating HCPs (regardless whether they did or did not attend a training session). The newsletters contained guideline recommendations, updates on the trial and relevant news items and information.

#### Interactive Website and Social Media

All HCPs gained access to an interactive website, containing information and guidelines for LBP, updates on the trial (also sent out via social media, i.e. Facebook and Twitter), and a forum on which they could discuss patient cases or other relevant issues.

### Data Collection and Analysis

This process evaluation among participating HCPs was based on several components developed by the Linnan and Steckler framework [3]. Data were collected using a mixed methods approach in which both quantitative and qualitative methods for data collection were applied (see Table 2). The subsequent paragraphs will describe the quantitative and qualitative data collection methods separately in more detail.

**Table 2:** Components of the process evaluation, their definitions, and data collection methods

Component	Definition	Data collection method
1. Recruitment	Procedures used to recruit HCPs	Description and minutes of recruitment procedure
2. Reach	Number of HCPs attending the training sessions as proportion of HCPs participating in trial	Registration at training session, minutes of research organisation
3. Dose delivered	Extent to which the protocol for the various strategy components was followed	Minutes of training sessions
4. Dose received	Experiences of HCPs with training session: satisfaction with individual components of training session and implementation materials Extent to which training content is applicable in practice of HCPs Extent to which HCPs expect to apply training content in practice, and extent to which HCPs expect the training to have effect in practice	Satisfaction questionnaires Minutes of training sessions and implementation process
5. Satisfaction	HCPs’ overall satisfaction with strategy	Questionnaires Qualitative interviews
6. Barriers and facilitators	Barriers and facilitators for collaboration and communication in clinical practice	Qualitative interviews Minutes of training sessions

*HCPs* health care professionals

### Quantitative

Immediately after the training session, HCPs were asked to fill in an anonymous questionnaire designed to elicit their satisfaction with the training session. Table 3 shows the various items of the questionnaire.

**Table 3:** Items of satisfaction questionnaire

1. Extent to which instructions are applicable in practice
2. Extent to which group composition was of benefit to learning process
3. Extent to which training methods were of benefit to learning process
4. Extent to which FlashBack video was of benefit to learning process
5. Extent to which discussion about barriers and facilitators were of benefit to learning process
6. Extent to which role play was of benefit to learning process
7. Extent to which educational material was of benefit to learning process
8. Overall rating of training session in terms of satisfaction
9. Expected effectiveness of training session on guideline adherence in practice
10. Expected extent to which instruction will be applied in practice by HCPs

*HCPs* health care professionals

All items other than items 8 and 10 were rated on a 6-point Likert Scale ranging from 1 (very bad) to 6 (very good). Item 8 was rated on a scale of 0 (lowest satisfaction) to 10 (highest satisfaction) and item 10 was rated on a 5-point Likert Scale (1. Always, 2. Frequently, 3. Regularly, 4. Sometimes, 5. Not at all).

The completed questionnaires were entered into 2 individual SPSS datasets. To ensure correct entry of the data, the two datasets were compared to each other using a DIFF function in IBM SPSS Statistics 20.0. Wrong entries (1.2 %) were manually checked and corrected to result in 100 % correct data entry. Quantitative data were analysed using descriptive statistics in SPSS 20.0.

### Dose Delivered

Dose delivered is the extent to which the protocol was followed. The various strategy components of the protocol are described below. For every component, the sum of individual training scores was used to calculate dose delivered per component and overall dose delivered.

#### Multidisciplinary Continuing Medical Education

Each training session included 5 components, which each received 1 point if executed as planned, with a maximum of 5 points (=100 % delivered) per training session.

#### Take-Home Educational Material

Take-home educational material was offered to all attending HCPs at every training session. Every session at which this material was offered received 1 point, with a maximum of the total number of training sessions provided.

#### Rules of Conduct and Contact Details

Every training session was followed by an e-mail to all HCPs in the respective clusters, containing the rules of conduct (1 point) and contact details of all HCPs (1 point). The e-mail was sent within 1 month following the training session. For every training session 2 points were received if the e-mail contained both components, and 1 point if only one of the components was sent. Thus, a training session could receive a maximum of 2 points for these components.

#### Quarterly Reminders

Every training session was followed by quarterly reminders (by e-mail) to all HCPs in the respective clusters. The reminders were sent at 3, 6, and 12 months after the training session. Every training session could receive 1 point for each time-point at which the reminder was sent out according to this protocol, and thus a training session could receive a maximum of 3 points.

#### Monthly Newsletters

Monthly newsletters were sent to all participating HCPs, commencing 1 month after the first training session had taken place, until follow-up of HCPs was completed (total of 20 months, from October 2014 to May 2016). For every month in which the newsletter was sent out 1 point was scored. The maximum number of points for this component was thus 20.

### Qualitative

In order to gain more in-depth knowledge on the satisfaction and experiences of the participants with the implementation activities, and to map barriers and facilitators for implementation of this guideline, semi-structured qualitative interviews were conducted with a subset of participating HCPs. The subset was chosen to represent all professions attending every cluster and training session with further interviews conducted until redundancy was reached (i.e. the point at which all topics were addressed repeatedly and no new topics emerged) [13]. Sampling of respondents was guided by availability of HCPs and their willingness to participate in an interview.

Interviews were partly based on the quantitative results of the satisfaction questionnaires and addressed satisfaction about the various implementation components. All interviews were analysed immediately afterwards using a constant comparison approach so that they could be used to guide subsequent interviews. In this way, both reliability and validity of data was enhanced. To further improve the validity and credibility of the data, member checking (a form of respondent validation in qualitative research) of summaries of interviews was completed with all interviewed HCPs, all of which agreed with the summaries and primary interpretations [14].

Interviews took place at a time and venue convenient for the HCPs. Due to booked schedules of the HCPs, most interviews were conducted by telephone. In these cases, all interviews were written down verbatim with pen and paper, put into orthographic transcripts, and subsequently typed into MS Word documents (‘transcripts’) immediately after the interview. In cases where interviews took place face to face, the interviews were audiotaped, and transcribed verbatim and summarized immediately after the interview.

The constant comparison approach was used to analyse interview data in three subsequent rounds. At first, transcripts were divided into descriptive and summarizing fragments (i.e. open coding). Secondly, fragments closely related to each other were grouped to gain provisional themes (i.e. axial coding). In the last step of data analysis, connections between the provisional themes were made and data were structured into meaningful entities relevant in the light of the interview aims [15]. Two independent researches coded data and any disagreements were resolved by consensus.

The analysis of the interview transcripts was compared to minutes of all training sessions to further heighten the quality of the findings by triangulation.

## Results

### Recruitment and Reach

The recruitment method led to the participation of 25 out of 41 GP practices involved in the network (response rate 61 %). The participating GP practices accounted for 53 individual GPs in this study, of which 31 attended the training sessions (GP reach 58.5 %). Furthermore, 19 out of 30 invited PT practices (response rate 63 %) participated in the study, accounting for 46 individual PTs, of which 42 attended the training sessions (PT reach 91 %). At last, 29 out of 100 invited OPs agreed to participate in the study (response rate 29 %), of which 23 attended the training sessions (OP reach 79 %). In total, 128 individual HCPs participated in this study. Ninety-six HCPs attended the training sessions, resulting in a total HCP reach of 75 %.

### Dose Delivered

#### CME

The HCPs were divided into one of four clusters. Due to cluster sizes, it was planned that the training would be delivered in two (for three clusters) or three (for one slightly larger cluster) separate sessions per cluster to ensure feasible group sizes and interaction between the participants (9 sessions in total). However, only 7 training sessions were performed as too few participants signed up for the other two sessions. Five of the 7 training sessions were executed as planned (5 points each). Due to technical issues, the video ‘FlashBack’ (second component of training) could not be shown in the other two sessions (4 points each). This yielded an overall dose delivered of 33/35 points, corresponding with an overall delivery percentage of 94.3 %.

#### Take-Home Educational Material

The protocol for take-home educational material was followed at all 7 training sessions, thus receiving 7 points (and 100 % dose delivered).

#### Rules of Conduct and Contact Details

The rules of conduct and social maps were sent out within a month of respective training for 5 of the 7 training sessions, while for the other two training sessions the documents were sent within 2 months. Thus, the overall delivery percentage was 71 % for these components.

#### Quarterly Reminders

The reminders were sent out according to protocol in 19 out of 21 time points. In the other 2 time points, the protocol was not followed timely, but the reminders were sent at a later moment. The overall delivery percentage for this component was 90 %.

#### Monthly Newsletters

Eighteen of 20 newsletters were sent. The other two newsletters were cancelled due to holiday seasons. The total dose delivered for this component was 90 %.

### Overall Dose Delivered

Overall, 87 out of 97 points were reached in dose delivery. All components taken together, the total dose delivered for this study was 89 %.

### Dose Received and Satisfaction

Ninety-one HCPs (94.8 %) that attended the training sessions completed the satisfaction questionnaire immediately following the training (Table 3). Table 4 shows HCPs’ overall satisfaction with the strategy. Nearly all components were rated as ‘good’ by most HCPs (presented as the Median). Exceptions were the rating of the ‘FlashBack’ video and the ‘Expected effectiveness’ of the training sessions in practice. These two components were mostly rated as ‘fairly good’. The range of the component ratings was high in most cases, indicating a wide data spread. Most HCPs expected to apply the contents ‘regularly’ into practice while the median of HCPs’ overall satisfaction with the study was 7.

**Table 4:** Results of satisfaction questionnaire

Item	N respondents	Median	Range (minimum–maximum)
1. Applicability^1^	91	5	2–6
2. Group^1^	90	5	3–6
3. Methods^1^	91	5	2–6
4. FlashBack^1^	59	4	2–5
5. Barriers and facilitators^1^	88	5	2–6
6. Role play^1^	91	5	1–6
7. Educational material^1^	79	5	3–6
8. Rating^2^	81	7	4–9
9. Expected effectiveness^1^	85	4	2–6
10. Expected application^3^	85	3	2–5

^1^Item could be rated on 6-Point Likert Scale ranging from 1 (very bad) to 6 (very good)

^2^Item could be rated on a scale of 0 (lowest) to 10 (highest)

^3^Item could be rated on 5-Point Likert Scale (Always, frequently, regularly, sometimes, not at all)

^4^Item could be rated on 5-point Likert Scale (1. Always, 2. Frequently, 3. Regularly, 4. Sometimes, 5. Not at all)

### Qualitative

Twenty semi-structured, qualitative interviews were conducted among HCPs who had participated in the training sessions. Seven GPs, 7 OPs, and 6 PTs were interviewed, of whom 8 were female and 12 were male. The data were analysed and categorised into 6 themes, discussed by theme below.

### Training Satisfaction

Overall, participants had positive experiences with the training sessions. The multidisciplinary and local character of the sessions were seen as positive factors. Some respondents indicated that they would appreciate a follow-up training session in one form or another, such as an interactive webinar, an annual seminar or regular short meetings. Respondents were positive about the content and methods of the training as well. Interactive methods were seen as conducive to learning, as was the role play. However, some participants mentioned they would have appreciated role play directed by actual patient cases from their own practice. In particular, discussion about practical barriers and facilitators for communication and collaboration were well received by participants, as it allowed to tackle their own practical issues that were relevant to them personally, and to hear what other disciplines thought about these issues. As one respondent illustrated: *“It was very instructive to talk to each other, to ask each other questions, to discuss with each other, to practice with cases, and to learn how everyone looks at those cases from his own perspective.”* (*GP, F, cluster 2*).

However, participants indicated that, although they appreciate all components of the strategy, they do not make (much) use of these resources (e.g. the website, social media, take-home educational material) due to time constraints.

### Multidisciplinary Approach

“*Multidisciplinary meetings and trainings are things that are rarely offered and done. In this sense, the training session had much value. It is important to discuss about cases and define them as a common problem*—*this is the only way you can truly work well together*.” (*OP, F, Cluster 2*).

Respondents indicated that multidisciplinary CME training sessions were not common practice, and that the multidisciplinary approach to this training was greatly appreciated. It allowed for people to get to know other professionals and exchange experiences with disciplines they hardly ever had contact with. For example, in current practice the OPs are not treated as part of the treatment team of a patient, while many patients do work and thus are treated, or at least seen by an OP if their LBP is long-lasting and results in impairments at work. Furthermore, participating in this training session resulted in better understanding of each other’s disciplines and perspectives. Respondents appreciated the opportunity to (learn to) look from the perspective of other disciplines and to learn about their professional role, expertise, capabilities, and knowledge. Finally it also allowed participants to exchange contact details, so that collaboration could actually take place in practice.

### Change of Attitude

Many respondents indicated that, although the training did not bring any new information regarding the treatment of LBP, the sessions were quite informative. Above all, it created awareness of the guideline and guideline recommendations, as one respondent stated: *“Stimulating the use of the same guidelines by all professionals is very good, in this way you can stimulate each other to be guideline adherent in practice, and you can tune treatment policy together.”* (*PT, M, cluster 1*).

The training clearly showed the added value of multidisciplinary communication and collaboration, which encouraged the use of the guideline. The training enabled participants to think about the benefits of communication and collaboration, and it opened the discussion about how to do this with each other. Furthermore, many respondents indicated using the patient as a messenger that would communicate a message from one professional to the other. The training session made them consider the possible pitfalls of using the patient as mailman, for example the way patients could wrongly interpret or communicate a message from one professional. *“The power of the training lay in our own unconsciousness: you learn to practice what you know, observe and interact with others. It is good to put yourself in someone else’s shoes for an evening.”* (*OP, M, Cluster 1*).

### Practical Barriers

Although participants appreciated the discussion about solutions to practical barriers, some indicated that they still did not have sufficient guidance and practical tips as how to overcome these barriers in practice. Some solutions, such as the development of a multidisciplinary back pain network or the integration of a daily collaboration moment, were very desirable, but not considered to be feasible to put in practice, because of personal, practical, or financial issues. This last category of barriers was considered the most important issue, since many professionals indicated that they only get paid for patient consultations, while interdisciplinary phone-calls, meetings, and time to conduct them, are not part of the payment deal provided by health insurers. This was cited as a reason for lack of willingness to collaborate actively. Another reported important practical barrier to efficient collaboration was medical confidentiality and the privacy of the patient. Patients have to give written consent for interdisciplinary exchange of their medical information. Usually this takes time, resulting in either delayed contact or failure to make contact at all. Practical barriers such as inaccessibility by telephone (due to, for example, different office hours of HCPs), lack of contact details of other HCPs, or complete unfamiliarity with the other HCPs of the patient, also play a role in failure to collaborate. According to all HCPs, unfamiliarity is particularly true for OPs, who often seem to be forgotten in the treatment of patients.

### Contextual and Organisational Barriers

While many participants indicated that they agree with the guideline recommendations and usually follow them, they noted that their attempts to practice guideline adherent care are increasingly being undermined by the recent rise of commercial centres for diagnostics and treatments. Moreover, guideline adherent care was considered open to interpretation, since all disciplines have their own guidelines and these are not always perceived to be in concordance with those of other disciplines. Furthermore, it appeared that LBP seems to be a problem that is now seen mainly in PT practice. Many professionals, however, were worried about the current health care system in the Netherlands, which pays HCPs per treatment, leading to the possible conflict between providing guideline adherent care and ensuring the continued existence of ones’ own health care practice.

### Confidence and Dependence

While many practical barriers to collaboration were identified, many HCPs mentioned confidence and dependence issues as a particularly important problems. Especially in the case of OPs, this played an important role. Professionals indicated that they are often distrustful of the OP, believing that he or she puts the interest of the company over the interest of the patient, as one participant put it: *“They* [*OPs*] *are in a difficult position where they often have to bite the hand that feeds them.”* (*GP, M, Cluster 2*).

Professionals believe that patients feel the same distrustful way about OPs. ‘Unknown makes unloved’ was a statement many participants made. Unfamiliarity and prejudice with each other led to the HCPs taking over each other’s roles in treating a patient, instead of making appropriate use of each other’s expertise. Therefore, familiarity and knowledge about each other’s’ roles and expectations were highly desired by most HCPs.

## Discussion

One hundred and twenty-eight HCPs participated in the implementation study, of which 96 participated in quantitative and 21 participated in qualitative evaluation. Overall dose delivered for this study was 89 %, and the participants were satisfied with the strategy, mostly with the multidisciplinary approach, which contributed to the mutual understanding of each other’s disciplines and perspectives. While the training sessions were not perceived to have yielded any new information, the strategy created awareness of the guideline and its recommendations, contributing to positively changing attitudes and aiding in improving guideline adherent behaviour. However, many barriers to implementation were still identified, including personal and practical factors, confidence, dependence and distrust issues among the HCPs, as well as policy factors (e.g. reimbursement systems).

The recruitment method applied in this study is considered successful, since it resulted in the randomization of 128 HCPs in the study. The reach of this study was as high as almost 77 %. Of the attendees, 32 % were GPs, 44 % were PTs and 24 % were OPs. This distribution allowed forming multidisciplinary clusters and training sessions, in which the main message of the guideline, i.e. more collaboration and communication between professional groups, could be well discussed and practiced. All but one training session included attendees from all three professions. Furthermore, the calculated dose delivered of 89 % is considered high for the current complex study. A review performed by Durlak and DuPre [2], showed that near-perfect implementation is unrealistic, and that positive results of implementation studies have often been obtained with levels around 60 %. Few implementation studies have attained levels greater than 70%, and 100 % implementation for all providers was not documented in any of the studies reviewed by Durlak and DuPre [2]. However, as there are no standardized formulas to calculate dose delivered, these results should be interpreted with caution, since they may be overestimations of the true dose delivered. The fairly simple method of calculating individual delivery scores by assigning points to various items and adding them up might not fully do justice to the complex relationship between the strategy components, which are probably not all equally important in changing HCP behaviour.

The context in which this study was conducted might be one explanation for the difference in reach of the various professions, as the PTs were the group most attending the training sessions, and OPs were overall in minority. In fact, GPs and PTs indicated that the past few years, patients with LBP are increasingly making use of PT facilities, rather than visiting their GP. This probably is the result of the previously mentioned introduction of a new health care system in the Netherlands in 2006. A study performed a year after the introduction of direct access to physical therapy showed that patients who reported having back pain were more likely to use direct access for a PT visit than patients with other symptoms [16]. Furthermore, back pain is the most commonly reported symptom by patients visiting a PT in the Netherlands [17]. A more recent study published by Scheele et al. [18] reported similar results and showed that the percentage of back pain care episodes for which patients directly accessed their PT increased substantially from 28.9 % in 2006 to 51.2 % in 2009. The high attendance rate of PTs in the current study compared to the attendance rates of GPs and OPs is in line with these findings.

Participants were satisfied with the training offered, and overall expected the training to be effective in daily practice for adhering to the guideline recommendations. Furthermore, participants expected to apply the methods learned during the training in daily practice. The interviews, conducted among a subset of these HCPs, indicated that the fact that this CME training was multidisciplinary and locally organised makes the application of the recommendations of the guideline into daily practice easier. This is in line with previous studies, which have shown that multidisciplinary approaches are (cost-)effective in reducing pain, disability, and fear avoidance beliefs, and that they improve work status, functional recovery and quality of life of patients with low back pain [19, 20]. The multidisciplinary and local approach allowed professionals to get acquainted in an informal manner and have the opportunity to familiarize themselves with the perspectives of other involved HCPs. Furthermore, a multidisciplinary approach stimulates mutual understanding and thus better communication. This is especially important for patients with LBP, who frequently have reported poor communication and collaboration between HCPs to be an important barrier to recovery, as it leads to conflicting treatment advices and poor coordination of care [10, 21].

Despite the HCPs’ satisfaction, positive experiences, and intentions to adhere to guideline recommendations in daily practice, actual adherence may nevertheless not be achieved, as many barriers still exist. As the current process evaluation has shown, these barriers include personal, practical, and financial factors. Most of these barriers have a practical nature in which time limitations, unfamiliarity with each other, and lack of guidance and reimbursement play a major role. Many of these factors have been found in earlier research as well, and are described in more detail in a review performed by Cabana and colleagues in 1999 [22]. This is quite worrisome, as it indicates that barriers for collaboration have not changed in over 15 years, and one therefore may wonder if improvement can ever be expected. Furthermore, other professionals who were not involved in this study but collaborate with the participating HCPs may not have been aware of the guideline recommendations. Up to now, most studies aiming to implement LBP guidelines by means of educational strategies involved monodisciplinary activities and have shown modest effects on guideline-adherent behaviour [8, 23–25]. Few studies have reported process outcomes. French et al. [9] investigated the fidelity of their implementation efforts and found that their intervention was delivered with high adherence to their implementation protocol. One study from 2003 that applied a multidisciplinary approach (i.e. physicians and nurse practitioners/physician assistants) found increase in guideline-adherent behaviour, but also reported poor adoption of implementation materials and methods [26]. These findings might suggest that the effectiveness of implementation strategies on actual practice thus depends not only on the provided strategy and the efforts of the HCPs within one study, but also on the collaboration with other professionals outside the study context. By involving several HCPs in a multidisciplinary and interactive approach, the current study aims to broaden the reach and uptake of the implementation activities, and thereby improve guideline adherence in various professions.

## Strengths

Immediately after a training session, participants completed satisfaction questionnaires, which were analysed shortly after their collection. To gain more in-depth understanding of the results of these questionnaires, a subset of HCPs from every training session were interviewed. The interview data were analysed directly after the interview, and quantitative and qualitative data were combined and used to improve the training for the subsequent sessions. Using this constant comparison approach was a strength that allowed for the adaption of the implementation strategy to the participants’ preferences and practice routines [2], although this led to the delivered doses of the current study not reaching 100 %. The fact that all three HCP professions were represented in quantitative as well as qualitative data collection is a further strength of the current study. The triangulation of data collection methods and the applied member checking further improved validity and reliability of the results of this process evaluation [14].

## Limitations

Some limitations have to be taken into account when interpreting the results of the current study. Firstly, the operationalization of the quantitative process outcomes has been done during the trial, and was thus not published in detail in the study design article. Secondly, this evaluation might overestimate the positive experiences of the participants due to selection bias. HCPs who participated in this study and actually attended the training session are more likely to be motivated for and inclined to change. For the same reason, selection bias might especially be relevant for the interpretation of the qualitative data collected among a subset of HCPs that attended the training sessions. A further pitfall in the data collection method might be the pragmatic approach to qualitative data collection among some of the interviewees due to time restraints. For example, some respondents were not able to schedule more time for the interview than the standard time for one patient consult (i.e. 10 min). Due to this limited time to interview these particular respondents, not every topic could be discussed. Furthermore, this time limitation might have resulted in respondents not sharing all their thoughts about the discussed topics. By interviewing other respondents more in-depth, and conducting interviews until redundancy was reached, an effort was made to reduce the limitations posed by this pragmatic approach. It has to be noted that part of the qualitative interviews were conducted by telephone, while others were conducted face-to-face. While interviews were audiotaped in most cases, and always transcribed immediately after the interview had taken place, it raises the question whether the interview types are of equal quality. Efforts were taken to limit this issue by following the same protocol in both types of interviews, and conducting member checks to allow for rectifications in misinterpretations or additional comments. At last, as mentioned previously, the calculated scores presented in the current paper should be interpreted with caution. It is highly recommended but challenging that measures be developed that make it possible to calculate informative implementation scores, such as doses delivered, in which complex relationships between strategy components, and the weight and importance of the various components are accounted for.

## Conclusion

The data presented in this paper have shown that the current strategy is feasible for implementation of the guideline in a Dutch primary care setting. However, the results of this study might not be generalizable to other countries given the specific health care context in the Netherlands. Furthermore, wide scale implementation is subject to several conditions, which include addressing practical barriers to change, such as time limitations, which can be changed by the involved professionals. More challenging barriers for guideline adherence, such as reimbursement systems of health care insurers, are beyond the control of the professionals as well as researchers aiming to implement a guideline. These barriers, not limited to the Dutch setting, have to be addressed on a policy level in order to allow for implementation of guidelines. As noted, these barriers seem to exist for almost 2 decades, and resolving them should be a high priority for those concerned with reducing the rising health care costs in many countries. The feasibility of large-scale implementation of a guideline using the presented implementation strategy must be weighed against the results of the cost-effectiveness of this strategy.
